# Healing war wounds and perfuming exile: the use of vegetal, animal, and mineral products for perfumes, cosmetics, and skin healing among Sahrawi refugees of Western Sahara

**DOI:** 10.1186/1746-4269-8-49

**Published:** 2012-12-27

**Authors:** Gabriele Volpato, Pavlína Kourková, Václav Zelený

**Affiliations:** 1 Department of Social Sciences, Wageningen University, Wageningen, The Netherlands; 2 Department of Landscape Architecture, Faculty of Agrobiology, Food and Natural Resources, Czech University of Life Sciences, Prague, Czech Republic; 3 Department of Botany and Plant Physiology, Faculty of Agrobiology, Food and Natural Resources, Czech University of Life Sciences, Prague, Czech Republic; 4 Via Montagna Grande 4, Montebelluna, TV, 31044, Italy

**Keywords:** Ethnobiology, Ethnobotany, Medicinal plants, Western Sahara, Sahrawi refugees

## Abstract

**Background:**

Over the past decade, there has been growing interest within ethnobiology in the knowledge and practices of migrating people. Within this, scholars have given relatively less attention to displaced people and refugees: to the loss, maintenance, and adaptation of refugees’ ethnobiological knowledge, and to its significance for refugees’ wellbeing. This study focuses on cosmetics and remedies used to heal skin afflictions that are traditionally used by Sahrawi refugees displaced in South Western Algerian refugee camps.

**Methods:**

The research methods included a structured survey carried out with 37 refugee households, semi-structured interviews with 77 refugees, 24 retrospective interviews with refugees and other knowledgeable informants, and a voucher specimen collection of the plants and products cited.

**Results:**

We recorded the use of 55 plant species, nine animal species, and six mineral products used within the three main use categories discussed in this paper: 1) Remedies for health issues that are typical of the desert environment where the Sahrawi once lived as nomads and now live as refugees (e.g. eye afflictions); 2) Remedies for wounds that are influenced by the Sahrawi’s recent history of guerrilla warfare; and 3) Cosmetics and products used for body care, decoration and perfuming (e.g. hair care, teeth cleansing, *henna* use) and for aromatizing the air inside of tents and which are widely used in everyday life and social practices.

**Conclusions:**

We discuss the changes that have occurred in the patterns of use and procurement of these products with exile and sedentarization in refugee camps, and conclude that refugees are not simply passive recipients of national and international aid, but rather struggle to maintain and recover their traditional ethnobiological practices in exile. Finally, we suggest further research into the ethnobiological practices and knowledge of displaced populations.

**Resumen:**

Sanando las heridas de guerra y perfumando el exilio: el uso de productos vegetales, animales y minerales con fines de perfumería, cosmética y curativos de la piel entre los refugiados saharauis del Sáhara Occidental.

**Antecedentes:**

Durante la última década ha habido un creciente interés en los estudios etnobiológicos de los conocimientos y prácticas de las personas que migran. Dentro de esta tendencia, los estudiosos han prestado relativamente menor atención a las personas desplazadas y a los refugiados: a la pérdida, el mantenimiento y la adaptación de sus conocimientos etnobiológicos, y su importancia para el bienestar de los refugiados. Este estudio se centra en los cosméticos y remedios para curar problemas de la piel tradicionalmente utilizados por los refugiados saharauis en los campamentos de desplazados al sudoeste de Argelia.

**Métodos:**

Los métodos de investigación que se utilizaron son: una encuesta estructurada con 37 familias de refugiados, entrevistas semi-estructuradas con 77 refugiados, 24 entrevistas retrospectivas con refugiados e informantes conocedores, y una colección de muestra de las plantas y productos citados.

**Resultados:**

Se registró el uso de 55 especies vegetales, nueve especies animales, y seis productos minerales utilizados en tres principales categorías de usos: 1) Recursos contra los problemas de salud característicos del entorno desértico donde una vez vivieron los saharauis como nómadas y donde ahora viven como refugiados (por ejemplo problemas en los ojos); 2) Remedios para heridas que reflejan la historia reciente de guerra de guerrilla de los nómadas saharauis; y 3) Cosméticos y productos utilizados para el cuidado del cuerpo, decoración y perfumes (por ejemplo, atención al cabello, limpieza de dientes, uso del *henna*) y como aromatizantes del aire al interior de las tiendas, que son ampliamente utilizados en la vida y las prácticas sociales cotidianas de los refugiados.

**Conclusiones:**

En la discusión, se analizan los cambios que se han producido en los patrones de uso y en la adquisición de estos productos durante el exilio. Llegamos a la conclusión de que los refugiados no son recipientes pasivos de la ayuda nacional e internacional, sino más bien luchan para mantener y recuperar sus prácticas tradicionales etnobiológicas en el exilio. Finalmente, sugerimos nuevas direcciones para la investigación de las prácticas y los conocimientos etnobiológicos de las poblaciones desplazadas.

## Introduction

Over the past decade, there has been growing interest within ethnobiology and especially ethnobotany studies in the knowledge and practices of migrating people [1-5]. Within this, scholars have paid relatively less attention to displaced people and refugees: to the loss, maintenance, and adaptation of refugees’ ethnobiological knowledge, and to its significance for refugees’ wellbeing and culture [6-9]. Specifically, the nomadic heritage and ethnobiological knowledge and practices of the Sahrawi people of Western Sahara have been largely overlooked in academic research over the past three decades. This is largely attributable to the effects of the Moroccan-Polisario war, which was fought over the control of Western Sahara, as well as to the overwhelming attention to the crisis situation that refugees confront, as the Sahrawi were forced to sedentarize in camps in Southwestern Algeria. With the end of military confrontations about 20 years ago, a window of opportunity for fieldwork has opened as logistical conditions have become more favourable, and some scholars have begun to carry out anthropological research among Sahrawi refugees and nomads. For example, Volpato et al. [10,11] investigated refugees’ use and procurement of medicinal plants, and Cozza [12] addressed the significance of food for Sahrawi refugees’ cultural identity. There is very little published on the Sahrawi’s use of plants for skin healing, cosmetic and perfuming purposes. Useful information can be found in a recently published study on plant uses in Western Sahara [13], as well as in certain historical sources from the colonial period [14-16]. This paper will therefore contribute to the knowledge of Sahrawi ethnobiology, offering a case study of refugees’ ethnobiological practices regarding medicinal and cosmetic products.

Humans have used vegetal, animal, and mineral products for cosmetic, perfuming, and skin care and healing for thousands of years [17-21]. In ethnobotanical studies, few publications have addressed the traditional use of plants for skin healing, as cosmetics, and for hygienic and perfuming purposes [20,22-24]. Following Aburjai and Natsheh [25], we define *cosmetic products* as substances or preparations intended to be applied to the external parts and mucosas of the human body in order to clean, perfume, or protect them, or to change their appearance. Besides products for skin healing, in this paper we include also products that the Sahrawi use for injuries such as snakebites and scorpion stings, for sensory system disorders such as eye and ear afflictions, and for afflictions of the upper part of the digestive system, namely lips, gums, teeth, and mouth. We exclusively focus on external applications, which include dressings, liniments, lotions, drops (e.g. eye drops), fumigants, plasters, and washes (e.g. mouth washes).

We present and discuss data about: (1) the use of products for skin afflictions and healing; (2) the use of products for cosmetic, aromatizing, and perfuming purposes; and (3) the origin and procurement of these products in a refugee context.

## Background

**Figure 1:**
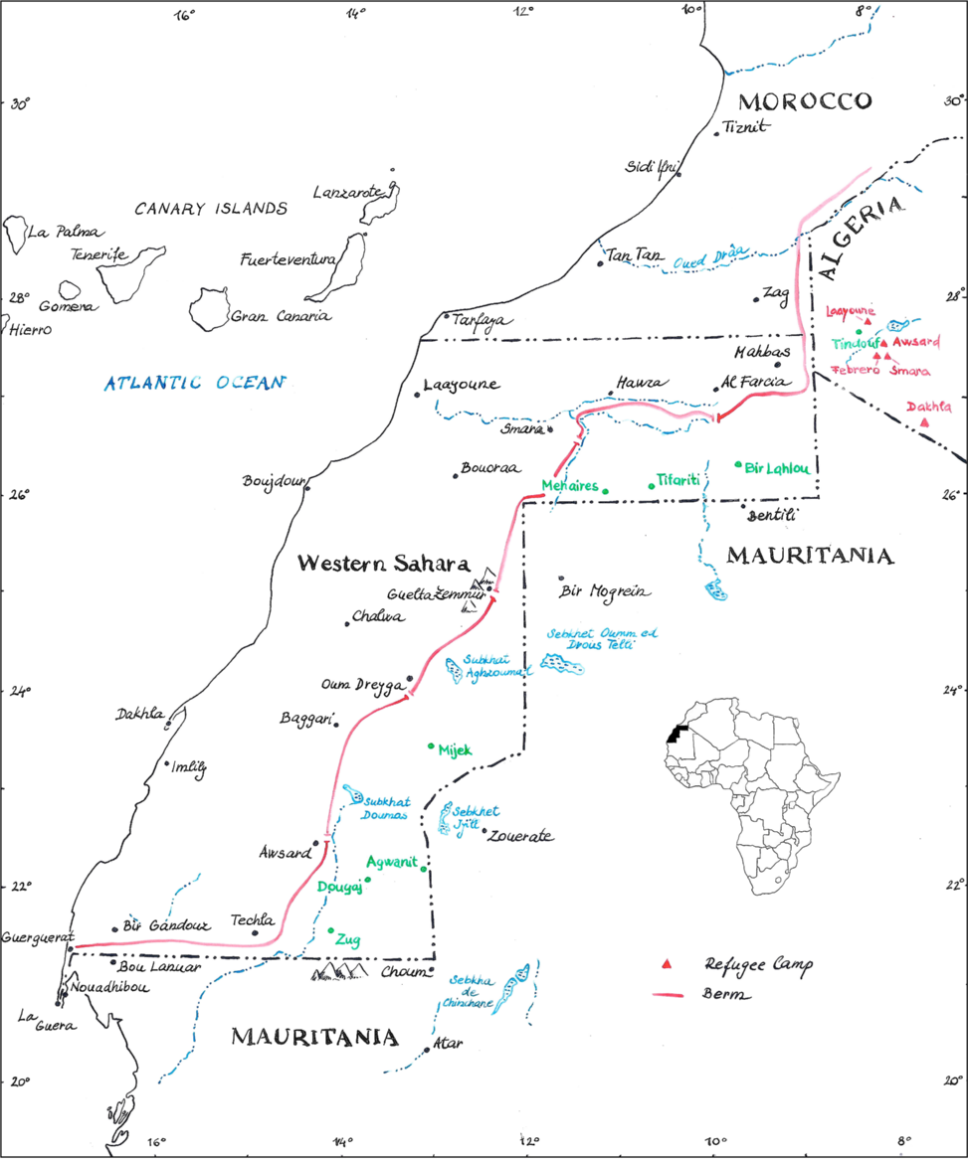
Map of Western Sahara and the study area (author: P. Kourková).

**Figure 2:**
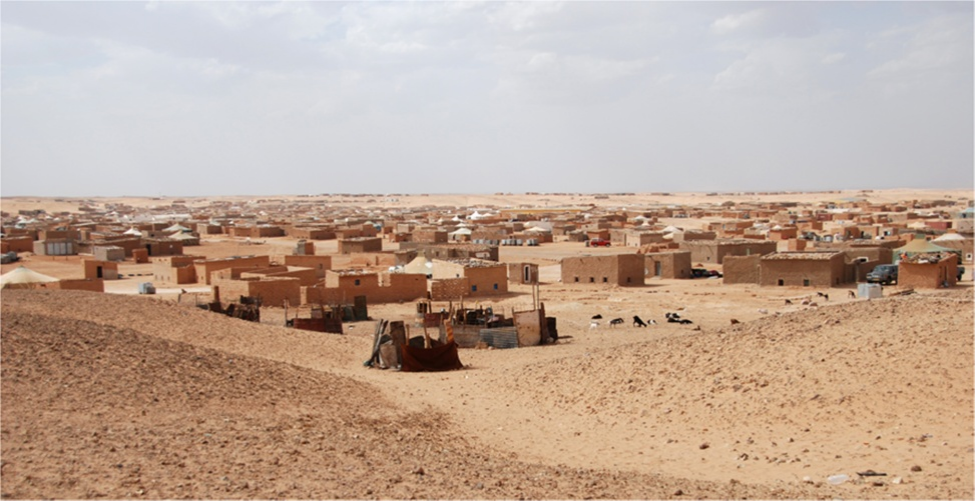
Sahrawi refugee camp (G. Volpato).

‘Sahrawi’, literally ‘people from the desert’, is the name given to nomadic and pastoral tribes that traditionally inhabited a coastal area of Northwestern Africa that includes Western Sahara, Northern Mauritania, and part of south-western Algeria. The Sahrawi people were essentially nomadic, raising camels, goats, and sheep in the rocky and sandy low-lying plains of Western Sahara, and relying on camel milk and meat, and traded their livestock for dates, sugar, cereals and legumes in markets on the periphery of their nomadic areas [15,26,27]. In 1975, after fifty years of Spanish colonial rule and following Morocco’s occupation of Western Sahara, about 70,000 Sahrawi had to flee the Moroccan army, and in the process became refugees [28,29]. After sixteen years of war (1975–1991) between Morocco and the Polisario Front, Morocco constructed and militarily defended a wall that cuts across Western Sahara in a North–south direction, excluding the nomads and the refugees from the majority of their Western Saharan territory. Nowadays, some 165,000 Sahrawi live in four refugee camps located on a desert plateau called Hamada, near the Algerian city of Tindouf (Figures 1 and 2).

In the camps, refugees live in canvas tents and mud brick huts, experiencing severe problems with water and food supplies, where car batteries are their main source of electricity. The European Union, certain bilateral development arrangements, UN agencies, and several solidarity groups provide food, shelter, and other basic commodities for the refugees [29]. Over the years, in the search to improve the quality of life in the camps, refugees have developed an informal economy that includes marketing of many products (from clothes to personal hygiene products and food items that supplement the diet provided by humanitarian assistance), expanding camp trading routes with Senegal, Mali, Mauritania, Algeria, and Spain, where the majority of the Sahrawi diaspora reside [28,30,31]. In the process, they have also reactivated both social and market networks for procuring traditional ethnobiological products [10].

Aside from its control over the camps, the Sahrawi’s political representative, the Polisario Front, also has political control over the eastern part of the Western Sahara, which was recovered from Morocco through guerrilla warfare that ended only with the peace agreement of 1991 [31]. These inland areas of Western Sahara are the so-called ‘liberated territories’ (approximately 20 per cent of the Western Sahara), while the remaining ‘occupied territories’ remain under the administrative control of the Moroccan government. Within the liberated territories, pastoral areas continue to be very important to the refugees as they attempt to maintain or recover their traditional livelihoods and cultural and social practices, e.g. from livestock husbandry to medicinal plant use [10,32].

## Methodology

Research on medicinal products used for skin healing was carried out in Sahrawi refugee camps between 2003 and 2007 (Fieldwork A), and research on cosmetics and perfuming products was conducted over a two-month period between March and April 2008 (Fieldwork B). The research methods for the ethnobiological analysis included semi-structured and retrospective interviews with refugees and informants regarded as especially knowledgeable, a ‘walk in the woods’ approach conducted with these knowledgeable informants, and a voucher specimen collection of the plants and products cited [33-35]. The semi-structured interviews collected data concerning: products used for cosmetic and perfuming purposes (i.e. to prepare perfumes and to aromatize the environment, to wash hair, skin, and clothes) and for skin afflictions; parts used; names in Hassaniya (the Berber-influenced Arabic language spoken by the Sahrawi); type of preparation and use; purpose of use; place of procurement; and the ecological status of the plants and animals used (if wild or cultivated/domesticated). The retrospective interviews were conducted with older informants and were aimed at understanding these products’ patterns of use in pre-war nomadic livelihoods.

Regarding Fieldwork A, a survey of 37 refugee households was administered to the person in the household more knowledgeable about skin healing and medicinal remedies (mean age of 56 ranging from 26 to 84; thirty-three of them being women and four men), and 45 semi-structured interviews and 16 retrospective interviews were conducted with refugees about the use and procurement of traditional medicinal remedies. Regarding Fieldwork B, semi-structured interviews were conducted with 32 informants in the refugee camps (Aaiun, 27 de Febrero, and Smara), and retrospective interviews were conducted with six informants; respondents had a mean age of 58 (ranging from 34 to 82), and all were women, as they are the holders of knowledge of cosmetics in refugee households [36].

In both field studies, interviews were conducted in Hassaniya, recorded, and translated into Spanish by local research assistants (the first author is fluent in Spanish, which is the Sahrawi’s second language, spoken since the time that the Western Sahara came under Spanish colonial control). In each case, prior informed consent was obtained verbally before the interview was conducted and before a camera or voice recorder was used. The study aims, methods, and outputs were explained. Throughout both field studies, the ethical guidelines adopted by the American Anthropological Association [37] and by the International Society of Ethnobiology [38] were followed, and specific methodological and ethical advice for research with refugees was taken into consideration [39].

Botanical species cited by informants were photographed and collected, and the first author identified the species. Cultivated species (e.g. *Lawsonia inermis*) were not collected. Some species could not be found in the field and so were identified through photographs (e.g. *Convolvulus trabutianos*, *Neurada procumbens*). Animal and mineral products were photographed but not collected. Plant specimens were collected in the liberated territories and in the refugee camp areas, while dried specimens of the products cited during the interviews were obtained either by purchasing them in refugee camp shops or in Tindouf, or samples were donated by the interviewees. For some plants, identification was based on dried specimens and/or photos, so that identification was possible only at the genus level. For those plants that were only available as dried specimens so that species determination was not possible, informants were asked for local name as well as morphology and ecological characteristics of the plant, and these were then compared with literature [40-42] in order to identify a range of possible species. Photographs of these possible species were later shown to informants in order to identify the correct species (or at least their botanical genus). The identification of plant species through artifacts such as herbarium specimens, drawings, and photos has been recently addressed by Thomas et al. [43], who found that species identification by means of photographs was highly reliable. Plant nomenclature follows the Sahara and Western Sahara botanical standard treatises for Saharan species [41,42], and the International Plant Name Index (http://www.ipni.org) for all other species. Botanical names are written in full with author(s) and family name only in Table 1. Voucher specimens are currently available at the first author’s home address; they will be deposited in the National Herbarium of The Netherlands (Wageningen Branch – Herbarium Vadense) in February 2013.

**Table 1:** Vegetal species used by Sahrawi refugees as cosmetics and for skin healing

**Species (voucher number)**	**Family**	**Name of the plant in Hassaniya**	**Part used/Name of the part in Hassaniya**	**Preparation and way of use (way of use is topical application unless otherwise stated)**	**Use**	Way of procurement*	Place of procurement**
*Acacia ehrenbergiana* Hayne *(*1015*)*	Fabaceae	tamat	leaves	dried and powdered	antiseptic for wounds	C	badyia
			resin: el elk tamat	triturated, topical application to eyes	eye infections and conjuntivitis	C	badyia
*Acacia tortilis (*Forssk*.)* Hayne subsp*. raddiana (*Savi*)* Brenan var*. raddiana (*1010*)*	Fabaceae	talha	leaves, seeds: shumban	triturated	antiseptic for infected wounds	C	badyia
			resin: el elk talha	dried or roasted, triturated, topical application of a plaster obtained by mixing it with water, or alone in powder; a bandage is made and renewed for three days (‘because el elk is each day absorbed in the wound and disappears’)	snakebites, eye infection, to remove dirt from eyes, wound cicatriser, abscesses (el elk forces the abscess toward the centre and favours its maturation)	C/P	badyia, Tindouf market, refugee camp shops
			bark: dbag	triturated	wound cicatriser	C	badyia
*Allium cepa* L.	Alliaceae	besla	bulb	triturated	antiseptic for wounds	P	Tindouf market, refugee camp shops
*Allium sativum* L*.*	Alliaceae	thoum	bulb	triturated	antiseptic for wounds	P	Tindouf market, refugee camp shops
*Ammodaucus leucotrichus* Coss. et Dur. *(*1033*)*	Apiaceae	kammuna, kammuna t-rag	seeds	triturated, a plaster is made with water or fat, or applied as powder	snakebites, scorpion stings, infected boils (furuncles), to prevent infections in wounds (against ntaaf)	C/P	badyia, Tindouf market
*Anastatica hierochuntica* L. (1027)	Brassicaceae	kamsha	aerial parts	dried, triturated, boiled in water, topical application when cooled down/dried, triturated and mixed with water to form a plaster	to treat vitiligo and other white spots on the skin/mycotic skin infections, particularly on hands and nails	C	badyia
*Argania spinosa* (L.) Skeels	Sapotaceae	argan	seeds: bulez	oil obtained from the seeds applied as a cream	against aging and to hair to strengthen it	P	Morocco, Algeria (Bechar)
*Atriplex halimus* L. (1052)	Chenopodiaceae	legtaf	leaves, aerial parts	decoction, hair washes/decoction, applied as a plaster	the decoction gives a reddish tint used like henna for feet and hands/skin oedemas	C	badyia
*Balanites aegyptiaca* (L.) Del. (1086)	Balanitaceae	teichat	fruit: tug	roasted, peeled, an oily substance is extracted and applied to skin/the ashes obtained from burning the fruit are mixed with oil and applied topically/mouth washes with the infusion of fruit peels and leaves	to eliminate spots from the skin/mycosis/mouth infections	C	badyia
*Beta patellaris Moq.* (1075)	Chenopodiaceae	silk	seeds: habba silk	fried in oil, topical application in ears	otitis in children	C	badyia
*Calotropis procera* (Ait.) Ait. f. subsp. *procera*	Asclepiadaceae	tursha	stems	ashes from burning the stem	antiseptic and cicatriser for wounds	C	badyia, refugee camp area
			leaves	powdered, mixed with henna, application to hair	to make the colour of henna darker	C	badyia, refugee camp area
*Cassia italica* (Mill.) Spreng. (1020)	Fabaceae	fellajit	seeds	powdered, application to eyes	cataracts	P	Tindouf market
*Caylusea hexagyna* (Forssk.) M.L. Green (1031, 2068)	Resedaceae	dhenban	aerial parts	fresh, pounded, the juice extracted is mixed with oil or fat and applied topically to hair	washes to perfume hair and to treat lice, and a dressing with a cloth is made and left in place for 24 hours for hair loss and to stimulate hair growth	C	badyia
*Centaurea pungens* Pomel (1079)	Asteraceae	zreiga	leaves	triturated, application with oil	abscesses	C	badyia, Moroccan occupied territories
*Chamomilla pubescens* (Desf.) Alavi (1090)	Asteraceae	lerbien, uazuaza	flowering tops	pounded, application to aching tooth	toothache	C	badyia
*Cleome africana* Botsch. (1026)	Capparidaceae	lemkheinza, mkheinza	leaves	fresh leaves are cooked in camel hump fat and the resulting poultice is applied topically/triturated, a poultice of the fresh leaves is applied topically	wounds/toothache	C/P	badyia, Tindouf market
*Commiphora africana* (A. Rich.) Engl. (1017)	Burseraceae	dirs	stems, resin: umm nass	resin is triturated and applied topically/burnt in the tent	stems are used to clean teeth/as antiseptic for wounds and for skin infections/for perfuming and against evil eye	P	Tindouf market, Mauritanian markets and traders
*Convolvulus trabutianos* Schweinf. et Muschl.	Convolvulaceae	gandul	aerial parts	decoction	to apply to burns in order to avoid being left with a scar	C	badyia
*Corrigiola telephiifolia* Pourret (1089)	Caryophyllaceae	taserghinit	roots	dried and triturated/soaked in colonia	burnt in the fire in the tent to perfume the air/to prepare perfumes	P	Tindouf market, Moroccan occupied territories (El-Aaiún market)
*Cuminum cyminum* L. (1073)	Apiaceae	kammuna	seeds	triturated	infected wounds, skin infections	P	Tindouf market
*Cymbopogon schoenanthus* (L.) Spreng. (1087)	Poaceae	idkhir, liedjir	aerial parts	dried aerial parts are burnt, triturated, and applied	burns	C	badyia
*Cyperus rotundus* L. (1040)	Cyperaceae	sad	tubercules: tara, tharoub	dried and triturated	burnt in the fire in the tent to perfume the air; mixed with colonia water to make perfumes for hair and skin, and to perfume the traditional women dress (melhfa)	C/P	badyia, Tindouf market, refugee camp shops, Mauritanian traders
*Euphorbia calyptrata* Coss. et Dur. var*. involucrata* Batt. (1080, 2035)	Euphorbiaceae	rammadah	aerial parts	dried and triturated	skin infections and oedemas	C	badyia
*Euphorbia granulata* Forssk. (1055)	Euphorbiaceae	kbidet ed-dab	latex	topical application	snakebites	C	badyia
*Euphorbia officinarum* L. subsp*. echinus* Hook.f. & Coss. (1001)	Euphorbiaceae	daghmus, sharbout (when dry)	branches, inner part	a green branch is heated on the fire, cut open, and the inner part is applied topically	boils, abscesses, skin infections, toothache	C	badyia
*Ferula asa-foetida* L.	Apiaceae	antita	resin: antita	decoction, mouthwashes, or triturated and topical application/triturated in water and applied externally	toothache, protective of teeth/snakebites, to strengthen hair, to combat hair loss	P	Tindouf market
*Ferula communis* L.	Apiaceae	fasukh	resin	dried and triturated	burnt in the fire in the tent to perfume the air; mixed with water or colonia to perfume hair and to prepare creams for skin	P	Tindouf market, Mauritanian markets, The Middle East
*Hammada scoparia* (Pomel) Iljin. (1009, 1021)	Chenopodiaceae	remth	leaves	made into a poultice, mixed with water and inserted in the bite to ‘absorb’ the poison/triturated, infusion, washes/mouthwashes with the decoction	snakebites, scorpion stings/to wash hair and to combat dandruff; mixed with henna and oil and applied as a lotion to hair/’pulsant’ toothache, stomatitis, mouth infections	C	badyia
*Heliotropium ramosissimum* (Lehm.) DC. (2053)	Boraginaceae	lehbaliya	leaves	triturated/triturated leaves are mixed with oil to make a lotion applied to hair	burns, toothache, ntaaf/to make hair more shiny	C	badyia
*Launea arborescens* (Batt.) Maire (1071)	Asteraceae	umm lbena	latex	topical application	to eliminate warts	C	badyia
*Lawsonia inermis* L.	Lythraceae	henna	leaves	dried, triturated, mixed with warm water and applied	to dye hair, skin, and nails and perfume hair	P	Tindouf market, Mauritanian markets, refugee camp shops
			stem: mesuak	chewed and used as toothbrush	to clean teeth	P	Tindouf market, Mauritanian markets, refugee camp shops
*Lepidium sativum* L. (1060)	Brassicaceae	reshad	seeds: habb er shed, afatash	topical application to eyes	to eliminate dirt from eyes	P	Tindouf market
Lichen***		tenquilit	aerial parts: ergheta	dried and triturated	added to mixtures to put in the fire and perfume hair/mixed with colonia and other plants to make perfumes	P	Tindouf market, Mauritanian traders, Moroccan occupied territories (El-Aaiún market)
*Lycium* intricatum Boiss. (1085)	Solanaceae	ghardeq	leaves	decoction is made twice, left to cool for one day and then applied in drops	cataracts and eye inflammations	C	badyia
*Maerua crassifolia* Forssk. (1007, 1048)	Capparidaceae	atil	stem: mesuak	chewed and used as toothbrush	to clean and strengthen teeth, for cavities	C	badyia
			leaves: sadra el hadra	burnt, ashes are applied as powder or mixed with water in a poultice	cicatriser, antiseptic for wounds, boils, ntaaf, itching	C	badyia
*Mesembryanthemum cryptanthum* Hook. f. in Hook.	Aizoaceae	afzu	aerial parts	green aerial parts are pounded and mixed with water	used as soap for washing	C	badyia
*Neurada procumbens* L.	Rosaceae	saadan	leaves	dried, triturated, mixed with water, hair washes	to stimulate hair growth	C	badyia
*Nucularia perrinii* Batt. (1047, 2042)	Chenopodiaceae	askaf	leaves	fresh leaves are smashed and mixed with water to form a poultice	skin infections and wounds	C	badyia
*Pancratium trianthum* Herb*.*	Amaryllidaceae	amajij	flowers	as they are	women use the flowers for perfuming and adorning	C	badyia
*Panicum turgidum* Forssk. (1051)	Poaceae	mrokba, umm rekba	aerial parts	dried, triturated, a poultice is made with water	wound cicatriser, applied in the ear to kill insects that entered there	C	badyia
			roots	pounded, mixed with milk cream and applied topically for two days	head wounds, bone fractures	C	badyia
*Peganum harmala* L. (1066)	Zygophyllaceae	harmel	seeds	triturated and fried	to eliminate quists	P	Tindouf market
*Pergularia tomentosa* L.	Asclepiadaceae	ghalqa, umm el-jlud	leaves	dried, triturated and mixed with water	snakebites, scorpion stings, boils	C	badyia, refugee camp area
			latex	applied topically on a bandage	to eliminate warts and skin grains	C	badyia, refugee camp area
*Pistacia* spp.	Anacardiaceae	tidikt	resin	dried	added to colonia to prepare perfumes; burnt in the fire in the tent to perfume the air	P	Tindouf market, refugee camp shops, Mauritanian markets
*Rhus tripartita* (Ucria) Grande (1023, 1064)	Anacardiaceae	shdari	leaves, bark: dbag	dried and triturated, mixed with henna and water	to dye hair with a different tone from henna	C	badyia
*Rosa damascena* Miller	Rosaceae	ward	petals	dried	added to perfumed waters	P	Tindouf market
*Salsola imbricata* Forssk. (1054)	Chenopodiaceae	ghasal (‘the washer’)	aerial parts	crushed in the hands	used as soap to wash	C	badyia
*Salsola tetrandra* Forssk. (2020)	Chenopodiaceae	laarad	aerial parts	ashed and powdered	wounds, skin infections	C	badyia
*Salvadora persica* L. var. persica (1070)	Salvadoraceae	lerak	stem: mesuak	chewed and used as toothbrush	to clean teeth	P	Mauritanian markets and traders
*Salvia aegyptiaca* L. (1049)	Lamiaceae	tezouknit	fruits: afatash (‘the one that looks for something’)	applied to eye	cataracts (‘it prevents cataracts from growing’), glaucoma, to clean eyes from sand and dirt	C	badyia
*Santalum* spp.	Santalaceae	oud legmari	wood	cut or grated in small pieces	burnt in the fire in the tent to perfume the air, mixed with colonia to make perfumes	P	Libya, Egypt, Saudi Arabia
*Syzygium aromaticum* (L.) Merr. & L.M.Perry	Myrtaceae	qronfel	flower buds	dried and triturated	mixed with colonia to make perfumes for hair, creams for skin, and to perfume melhfas (melhfas are soaked in water with cloves during some days); a decoction of cloves is filtered and applied to hair to strengthen and perfume it, and to treat lice	P	Tindouf market, refugee camp shops
*Tamarix* sp. (1059)	Tamaricaceae	ar’ar	leaves	triturated, infusion or maceration in water, applied with a cloth	burns, sunstroke, especially in children	P	Tindouf markets, other Algerian markets (Algiers)
*Teucrium chardonianum* Maire et Wilczeck	Lamiaceae	shendgoura	flowering tops	dried and triturated	burnt in the fire in the tent to perfume the air; mixed with water and applied to hair to perfume it and stimulate hair growth	C/P	Moroccan occupied territories
*Ziziphus lotus* (L.) Desf. subsp. *saharae* (Batt.) Maire (1002)	Rhamnaceae	sdir	root bark	decoction	snakebites, poison antidote	C	badyia
*Zygophyllum gaetulum* Emberger et Maire (1050)	Zygophyllaceae	aggaya, el barraya (‘the healer’)	leaves	dried, triturated, heated on the fire, a plaster is made with water and applied in frictions	varices, snakebites, scorpion stings, and all skin infections	C	badyia, refugee camp area

* *C* collection; *P* purchase.

** Badyia is the term used by Sahrawi refugees to indicate the accessible part of their former nomadic areas. Here it includes the part of Western Sahara under Polisario control, North of Mauritania, and Southwest Algeria; Refugee camp area stands for the area in and around the refugee camps.

*** This lichen is known to informants only in its dried and triturated form; as a consequence, we were not able to identify it even at genus level.

## Results and discussion

The plant species used by the Sahrawi refugees for perfuming, cosmetic and skin-healing purposes are reported in Table 1 in alphabetical order according to botanical name. For each species identified, data reported include: botanical name, botanical family, voucher number, plant name in Hassaniya, part(s) used and name(s) of these parts in Hassaniya (if any), traditional preparation and means of use, and origin of the plants and/or products used (i.e. where informants harvested, bought or otherwise obtained the plant or plant part).

**Figure 3:**
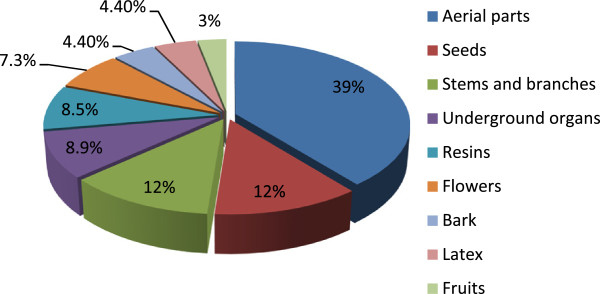
Plant parts used by Sahrawi refugees for perfuming, cosmetic and skin healing purposes.

A total of 55 species were reported. The species belong to 32 botanical families, where Chenopodiaceae (six species), and Apiaceae, Asteraceae, Euphorbiaceae, and Fabaceae (three species each) were the most representative families. The parts used are presented in Figure 3: aerial parts (leaves and non-woody stems) represent almost 40% of the total, followed by seeds (12%), woody stems and branches (12%), underground organs such as roots, bulbs, and tubercules (8.9%), and resins (8.5%). Nearly all of the products are dried before use or are purchased already dried: dry material accounts for 90% of the uses and in only 10% of the cases do the Sahrawi use fresh products. Fresh products are mostly obtained from readily available plants (either harvested from the local environment or purchased in the market) such as the aerial parts of *Caylusea hexagyna*, *Cleome africana*, and *Nucularia perrinii*. About 70% of the products are also triturated using a small mortar before use.

Regarding plant uses, about half refer to treatments for skin afflictions of various kinds (e.g. wounds, burns, and warts), snakebites and scorpion stings, and toothache and mouth afflictions. More than 40% of the total plant uses for skin afflictions are for the treatment of wounds (e.g. cicatrizers, antiseptic). Another 11% are used for eye and ear afflictions. Cosmetic uses account for about 20%, and aromatizing and perfuming uses for another 16%. About 35% of the total cosmetic and perfuming uses are for hair care. Two plants (3% of the uses) are used as soaps. Several plants have multiple uses and are used in both categories (e.g. *Lawsonia inermis*, *Hammada scoparia*), and could thus be categorized as *cosmeceuticals*[20,44].

Cosmetic and skin healing products of animal and mineral origin are presented in Tables 2 and 3 respectively. For each product the following data are reported: name in Hassaniya, preparation and means of use, purpose of use, and provenience. There are twelve products of animal origin (from nine animal species) and six of mineral origin, and all are used in topical applications. While animal-based products are mainly used for therapeutic purposes (e.g. wounds), mineral-based products are also important as cosmetics, particularly the widely used red hematite and galena.

**Table 2:** Animal derived cosmetics and products for skin healing used by Sahrawi refugees

**Ingredient**	**Hassaniya name**	**Preparation and way of use**	**Use**	**Provenience of the product**
Ambergris (from *Physeter macrocephalus* L.)	enebra	sun dried, triturated, topical application	snakebites	Tindouf market, markets of coastal Western Sahara
Camel (*Camelus dromedarius* L.) hump fat	ludek	heated and inserted in ears in drops and with cotton/heated, external application	otitis, earache/open wounds	refugee camps, badyia
*Cephalopina titillator* Clark (Oeastridae) - parasitic larva of camels’ nostrils and high respiratory ways	duda	larvae are squeezed into ears	strong earache in infants	refugee camps, badyia
*Chamaeleo* spp. (Chamaeleonidae) skin	buya	the dried skin is macerated in water and then applied topically for three days	boils, wounds, *ntiaff*, anti-venoms, toothache	Tindouf market, Mauritania
*Chamaeleo* spp. (Chamaeleonidae) egg	beid el buya	dried with salt or cooked, topical application	antidote for snakebites, to treat boils and skin abscesses	Tindouf market, Mauritania
Fennec (*Vulpes zerda* Zimmermann) bone	zib	boiled, triturated, topical application	herpes, measles	badyia
Goat (*Capra aegagrus hircus* L.) milk’s cream	zibde	heated and inserted as drops in ears or nose; or heated with barley, mixed with cooked dates and kept in a young goat’s leather (called *agreyia*); this product is known as *dhen*	otitis, earache; nosebleeds	refugee camps, badyia
Ostrich (*Struthio camelus* L.) egg*	naama	the shell is finely triturated and applied to eye/triturated to powder and sniffed	to eliminate ‘the white spot’ from eye (pinguecula)/nose bleeding	badyia
*Pimelia angulata* Fabricius (Tenebrionidae) - darkling beetle	anfusa	smashed, boiled in a small amount of water, topical application of drops	earache in infants	refugee camps, badyia
Urine		topical application	wound disinfectant**	
*Uromastyx acanthinura* Bell (Agamidae) skin	dab	the dried skin is macerated in water and then applied topically for three days	boils, skin infections***	badyia
*Uromastyx acanthinura* Bell (Agamidae) - kidneys, described as yellow fatty tissues	dab	boiled in water and inserted as eardrops	otitis and earache in infants and children	badyia

*species no longer present in the area.

**abandoned use.

***used in substitution of less readily available chameleon skin.

**Table 3:** Mineral and vegetal derived cosmetics and products for skin healing used by Sahrawi refugees

**Ingredient**	**Hassaniya name**	**Preparation and way of use**	**Use**	**Provenience of the product**
Charcoal		embers from an underground fire are cooled and applied externally	open wounds	refugee camps, badyia
Galena – Lead(II) sulphide (PbS)	kehla, kohl	scraped, topical application around eyes	cosmetic, to clean dirt from eyes, improve vision, treat glaucoma and cataracts	badyia
Potassium alum	shabba	put in a spoon on the fire until it becomes powdered, then mixed with water and applied topically around eyes/applied to a wound, kept in place with a cloth	to remove sand and dirt from eyes/wound cicatrizer and antiseptic (the pain concentrates toward one point until it disappears), snakebites and scorpion stings	badyia, Tindouf market
Red hematite, red ochre – Iron(III) oxide (Fe_2_O_3_)	hemera	scraped, topical application	around eyes to reduce solar radiation into eyes, to treat cataracts and conjuntivitis/on abscesses (all around the abscess) to stimulate them to mature, bone fractures (put at the junction of the break) and wounds	badyia
Red hematite	hemera tahia	same as above	same as above, but with enhanced power	badyia (but believed to come from meteorites)
Tar (of vegetal origin or bitumen)	gatran	applied on the face on the 27th of February	to darken face colour and scare the demons	refugee camps

The specific uses reported in the tables and outlined above reflect: 1) the more common health issues specific to the desert environment in which Sahrawi live (e.g. eye afflictions, snakebites); 2) the specific health issues that are characteristic of nomadic life and the recent history of guerrilla warfare (e.g. wounds); and 3) the specific cultural preferences and practices of the Sahrawi in terms of cosmetics and products used for body care, decoration and perfuming (e.g. hair care, teeth cleansing, *henna* use) and for aromatizing air inside their tents. Below, these main use categories are discussed, and then the origin of these products and the means by which Sahrawi refugees procure them are addressed.

### Eye and ear afflictions and snakebites

Like other populations living in desertic environments [45], the Sahrawi protected themselves from sun, sand and wind through loose body wraps and by covering their faces and heads with a veil or turban. In addition, women, especially but not exclusively, painted their eyelids with products that have both protective and sunscreen functions. Nonetheless, eye afflictions have always been one of the most common health problems encountered by the Sahrawi both as nomads [46] (pg 204) and today as refugees. For serious eye afflictions such as cataracts and glaucoma, the Sahrawi recurred to specialized healers, usually older women. These healers still practice in the refugee camps and use special treatments that at times consist of complex mixtures. For example, one healer reported using a mixture made with *Acacia* leaves, seeds of *Trigonella foenum-graecum* (one cooked and one raw), and a ‘bone of the *talha*’ (i.e. a bone left under an *Acacia* tree after a meal was consumed; the bone is burnt and triturated). This mixture is powdered, mixed with the blood of a spiny-tailed lizard or a young goat, and applied to eyes to treat cataracts (‘eyes progressively become white,’ an illness called *auar*). The patient is then advised to stay in a fresh and quiet place and to repeat the treatment for 40 consecutive nights.

Aside from such complex treatments, there are also common remedies found in the pharmacopoeia of Sahrawi families, the most well known being the fruit of *Salvia aegyptiaca*. This tiny fruit, called ‘afatash’ (which means ‘the one who searches’ because of its medicinal use) is applied to the eyes at night before sleeping, so it can ‘clean’ the eyes from sand and other unsanitary material. Scientific literature shows that it creates a mucilaginous coating and has the capacity to agglutinate these residues to its surface [11,47]. Some refugees report the same medicinal use for the seeds of *Lepidium sativum*, which are then also called *afatash*.

**Figure 4:**
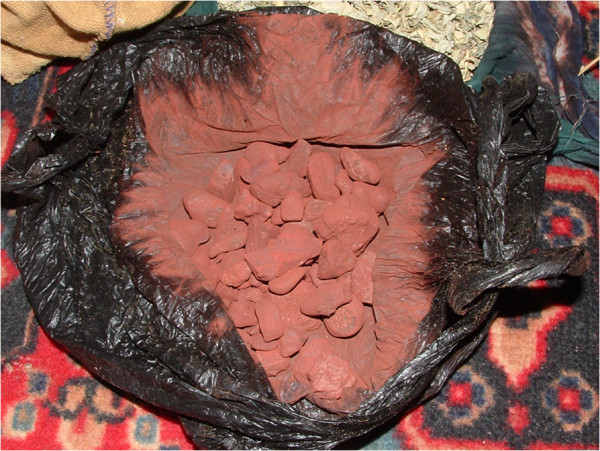
***Hemera *(P. Kourková).**

In addition, the Sahrawi widely use certain products of mineral origin to maintain or restore eye health. These are ground with a thin pointed iron and applied as eye cosmetics. Especially hematite and galena are used for these purposes, just as they have been used for millennia by the populations of North Africa and the Middle East as eye cosmetics and to treat eye diseases [48]. The use of red hematite (*hemera*, lit. ‘the red one’, Figure 4) to reduce the effect of solar radiation on the eyes and to treat cataracts and conjunctivitis deserves some notice, both because refugees commonly use it and because, according to informants, there are two types. The type that is regarded as most potent is red hematite that is believed to come from meteorites. Allegedly, these meteorites are collected in the desert, and distinguished from rocks of earth origin by their structure, relative weight, and spatial distribution that results from the fall from space.

Galena (*kehla* or *kohl*, lit. black) is mixed with fat to paint women’s eyes. It is applied cosmetically mostly by older women, but older men may apply it as well, especially tribal *sheiqs* and notables, as a sign of social standing. *Kehla* has been widely used by North African women for at least the past two to three thousand years; the practice of darkening the eyelids with galena is today encountered throughout Arabic countries and the Middle East for cosmetic, cosmeceutical and medicinal purposes, in spite of the fact that it may constitute a health hazard due to *kehla*’s lead content [49-51]. *Hemera* and *kehla* may also be mixed with triturated *Acacia* resin to obtain a mixture known as *acaz*, which is grated, filtered, and applied to the eyelids (or to their inner side) to improve vision.

Earache and otitis are other chronic health problems caused by the harsh desert environment, especially in infants and children. Infant earache is described as a pain or itching in the inner ear and adults detect it when infants cry and continuously scratch their ears. Refugees report the use of one vegetal product – the seeds of *Beta patellaris*, which are fried and applied topically – and of five animal-derived products to treat earache and otitis. Among the latter, fatty or fat-like substances are preferred; these include camel hump fat, goat milk cream, a yellowish substance found in the internal organs of *Uromastyx acanthinura* (perhaps the kidneys), the larvae of *Cephalopina titillator* (a parasitic fly inhabiting camels’ nostrils and upper respiratory system), and the darkling beetle *Pimelia angulata*.

Scorpions (i.e. *Androctonus australis* L.) and vipers (i.e. *Cerastes* spp.) are fairly common in the Western Sahara desert. A common snakebite treatment among the Sahrawi consists of making a series of cuts on the limb directly above the bite in order to draw out blood containing the venom before it enters into the circulatory system. In addition, animal and vegetal products (e.g. the latex of *Euphorbia granulata*, the aerial parts of *Hammada scoparia*, the root bark of *Ziziphus lotus*, chameleon eggs, and ambergris), all believed to ‘absorb the venom’ and halt its effects, are applied topically to the bite or sting site. Sahrawi uses of *Ammodaucus leucotrichus*, *Hammada scoparia*, and *Zygophyllum gaetulum* to treat snakebites and scorpion stings are not reported by Hutt and Houghton [52] and by Houghton and Osibogun [53] in their reviews and may deserve further attention.

### Wounds

Considering the high number of reports of antiseptic and cicatrizing treatments for wounds, it seems that they have been common among the Sahrawi. Besides everyday accidents (e.g. from *Acacia* spines, iron tool use, etc.), this may be related to the fact that Sahrawi nomads traditionally engaged in raids to procure camels. Inter-tribal wars to gain access to pasture areas and caravan routes were not infrequent, and the recent guerrilla war against the Moroccan Army implied a high number of wounds and injuries, and a consequently high number of treatments and treatment episodes. Both historical sources and Polisario soldiers’ memories of the war confirm this. Among Sahrawi nomads there were healers known as *tebib* who were called upon for life-threatening conditions; *tebib* who were specialized in treating fractures and wounds usually accompanied raiding and warring parties and would then take a share of the booty [15]. Sahrawi combatants in the Morocco-Polisario war continued the tradition using locally available resources to treat wounds in the battlefield, stressing the value of this knowledge and these practices in emergency situations.

**Figure 5:**
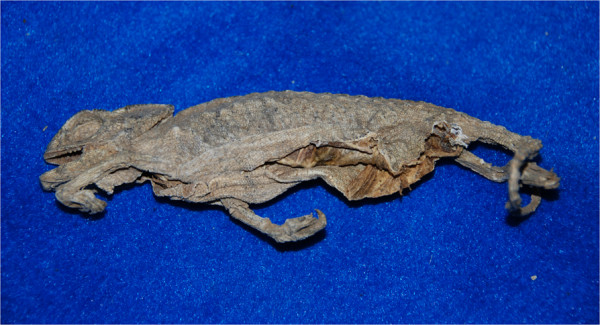
**Dried skin of chameleon, *el buya *(P. Kourková).**

The most widely reported products used to treat wounds are the resin of *Acacia tortilis* (*el elk*) and red hematite, which are also used in combination. *El elk* is collected during the hottest months from the trunks of *A. tortilis*; it is then dried and used for a variety of food and medicinal purposes [11,13,14]. The use of gum collected from *Acacia* species in topical application to treat skin infections and burns is reported among other African populations as well as in India [23]. Other vegetal products used as cicatrizers and antiseptics include resins from other species (e.g. *Commiphora africana*), tannin-rich bark and leaves of *Acacia* species and *Maerua crassifolia*, ashes obtained from *Calotropis procera* and *Salsola tetrandra* (Moors apply a poultice of ashes and water to treat itching and skin eruptions) [54], fresh leaves of *Cleome africana* and *Nucularia perrinii*, and the inner stems of *Euphorbia officinarum*, which is regarded as a panacea. Among animal-derived products, fat obtained from a camel’s hump is applied to open wounds, as is dried chameleon skin (Figure 5), or spiny-tailed lizard skin is used in substitution. Goat milk cream is mixed with the pounded roots of *Panicum turgidum* and applied topically to treat deep wounds and fractures. These latter and other products (e.g. the triturated seeds of *Ammodaucus leucotrichus*, or a mixture composed of barley, garlic, and onion, and called *tekelkuli*) are further used to treat *ntaaf*, which is described as an infected wound that can provoke fever, or as ‘a recurrence [of a wound or a sore] caused by a perfume’ [55] (pg 35).

### Cosmetic, aromatizing, and perfuming products

In Sahrawi culture, the importance of cosmetics and perfuming products has been reported historically in ethnographic studies on Western Saharan nomadic tribes. Gaudio [16] writes that ‘women like very much to perfume themselves with special essences and herbs coming from Mauritania, and often they dye their hair, eyes, hands and feet with mineral and vegetal substances’ (pg. 61), and Caro Baroja [15] reports the use of plants to perfume the bodies of the dead before burial (pg. 278). Among many populations, cleansing and perfuming plants are important in ritual ceremonies such as weddings and funerals [56]. The attribution of great cultural value to scent and perfume is widespread among Saharan nomads, e.g. the Tuareg [57]. After thirty years of exile, Sahrawi refugees continue to use traditional products for cosmetic and perfuming purposes. Older women in each household are responsible for storage, preparation, and use of these products, and they hold and transmit related knowledge and practices to younger female members.

### Aromatizing products

**Figure 6:**
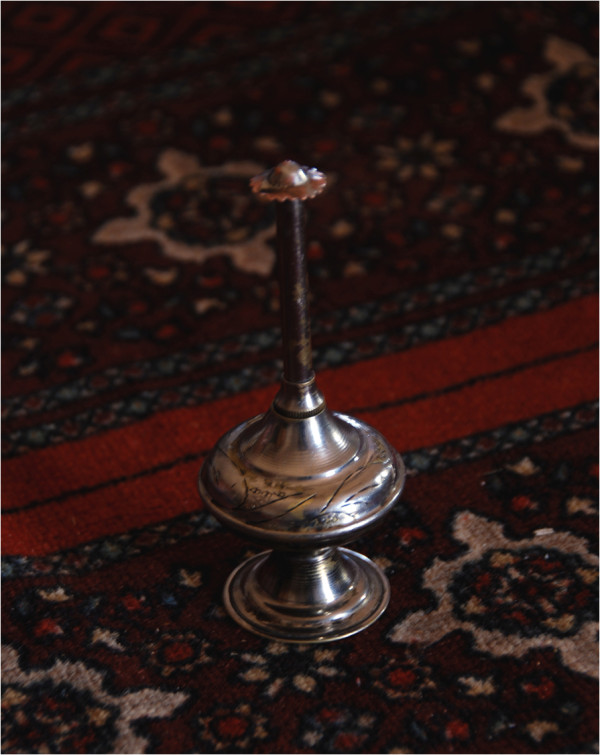
**Metal tool where incense is burnt, *lembarja *(P. Kourková).**

All refugee families keep a metal recipient inside the tent where they burn charcoal or wood to make tea and where they add aromatizing products in order to fill the tent with their fumes and smell. The most prized air aromatizer is the resin of *Pistacia* species, known by the Berber name *tidikt*. Small pieces of *tidikt* are put in the fire to perfume the air and are also added to scented alcohol-based formulations called *colonia*, where they dissolve rapidly, to prepare homemade perfumes that are also offered to guests. Other products burnt in the fire include the dried and triturated roots of *Corrigiola telephiifolia* and the dried and triturated tubercules of *Cyperus rotundus*, which contain essential oils used as aromatic therapeutics across North Africa [40,58]. These are all incorporated, along with sandalwood, common stick incense, powdered perfumed soap, and *Ferula* species’ resin, into a mixture called *lemhor*. Sahrawi women prepare *lemhor* and burn it in censers called *lembajra* (Figure 6).

**Figure 7:**
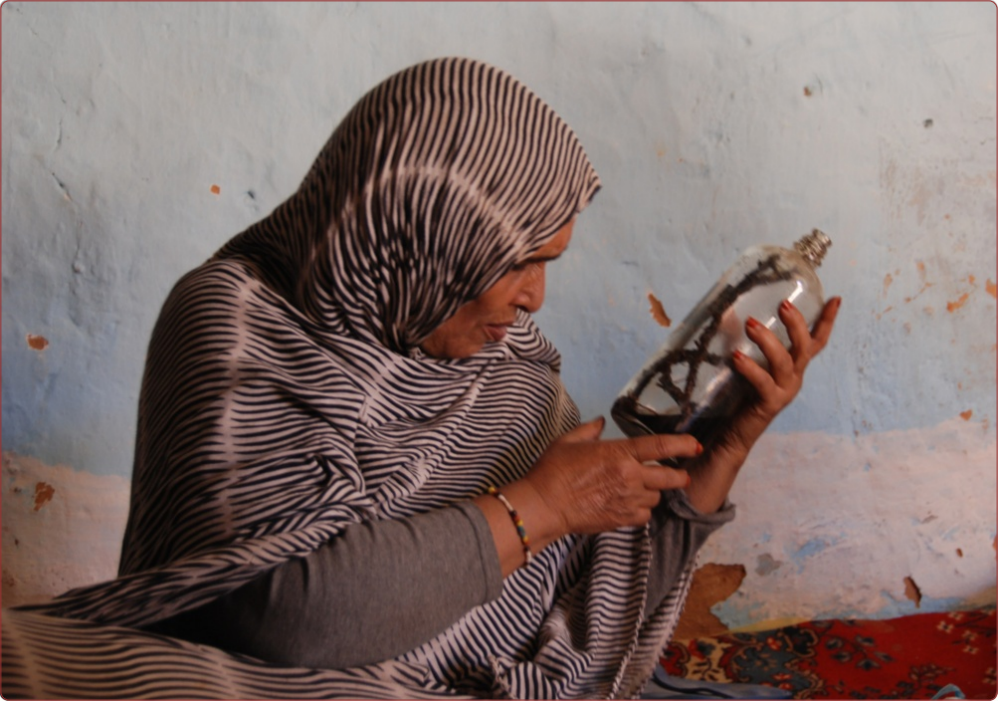
**Refugee woman with a bottle of hand-made perfume. *Corrigiola *roots are visible inside the bottle (P. Kourková).**

With the same ingredients and the addition of triturated cloves, *lemhor* is soaked in *colonia* and a perfume or a cream for hair and skin are prepared (Figure 7). *Tenquilit*, an unidentified species of lichen described as ‘a kind of green plant that grows on stones and trees in areas with water’, can be added. *Tenquilit* is commonly purchased in its dried and triturated form, called *ergheta*. Refugees explain that *tenquilit* is not itself aromatic but, when mixed with alcohol, it enhances other products’ aroma. Although scholars have paid little attention to their use among world populations, lichens have been used and traded since antiquity as food, medicine, decoration, and in dyes and perfumes [59-61].

As among the Tuareg [57], some plants burnt for aromatic purposes are also used in a spiritual context to remove the evil eye from affected persons and to treat ‘insanity’ by inhaling the fumes. The resin of *Commiphora africana* and of *Ferula* species are burnt for these purposes, at times together with pieces of pyrite, seeds of *Peganum harmala*, seeds of *Coriandrum sativum*, and pieces of the dried skin of *Chameleon* spp. To prevent evil eye in children, the same products are placed in a small leather bag, which is hung around the child’s neck.

### Cosmetics and hair care

Saharan and Sahelian women take much care when embellishing, perfuming, and adorning their hair [62]. Sahrawi women aesthetically value hair that is long, shiny, and perfumed. They use several plants to accomplish this: the juice obtained by pounding the fresh aerial parts of *Caylusaea hexagyna* is mixed with oil and applied to the hair to strengthen and perfume it, and for the same purpose the dried aerial parts of *Teucrium chardonianum* are macerated in water and the hair is then washed with the water. *C. hexagyna* may be used alone or in combination with *Neurada procumbens* to stimulate hair growth, and Barrera et al. [13] (pg 93) report a cosmetic use of *Acacia tortilis* that we did not collect: due to its pleasant aroma, women use the soft wood of old trunks, called *aharhar*, to perfume their hair. But by far the most important product in Sahrawi hair care is *henna*, the dried leaves of *Lawsonia inermis*.

*Henna* is a small tree native to the Middle East and Northwest India that, after humans introduced it into different parts of the world, is today present in many tropical and subtropical areas. At least since the time of the ancient Egyptians, it has been used as a growth accelerator, to combat hair loss, for decorating and dyeing hair, hands, and feet, and for the treatment of skin disorders, and it is an important plant especially among Islamic populations [25]. Sahrawi women widely use *henna* to embellish their bodies. The painting of hair, hands, and feet with *henna* is an important practice in different Sahrawi refugees’ social contexts, such as rites of passage, weddings, and as tribute to guests. For hair coloring, tone variation is obtained by adding other vegetal products to *henna*: for example, a darker color is produced by adding the leaves and bark of *Rhus tripartita* or, according to Barrera et al. [13] (pg 109), by adding a powder obtained from crushing *Calotropis procera* leaves. Sahrawi women use cloves for both medicinal and cosmetic purposes: they are pulverized and macerated in water to color and perfume the hair, also in combination with *henna*. A perfume made with cloves is used to perfume traditional women’s clothing – called *melhfa* – in important social gatherings such as weddings.

### Teeth cleansing

Stems from *Maerua crassifolia*, *Salvadora persica*, or *Lawsonia inermis* are used to clean teeth. These stems are known as *mesuak*, which the Sahrawi mainly collect from the first tree species. Stems are carefully selected, the bark is peeled off from one tip, and this is used as a toothbrush by passing it over the teeth, softly chewing it, or just keeping it in the mouth and playing with it. *Mesuak* are today widely used in refugee camps as they were in the past among nomads, and only refugees that have adopted a more Western lifestyle use plastic toothbrushes. The use of *mesuak* from *M. crassifolia* is also reported to improve vision, which may be due to the Vitamin A that its use provides. A *mesuak* is also often offered as a gift to guests who come to the home.

### Product origin and procurement

The Sahrawi refugees obtain cosmetic and skin healing products from plant species growing in the western Saharan environment (65% of the species), and those that are imported and purchased in markets, shops, or from traders (35%). The first category includes products from species such as *Acacia* spp., *Balanites aegyptiaca*, *Maerua crassifolia*, and *Ammodaucus leucotrichus*. These are also used by the Tuareg of Algeria, Mali, and Niger [63] and by Moor populations of Central Mauritania [54], among other African pastoral groups. In the second category are plants used in the perfume industry (e.g. *Rosa damascena*, sandalwood) or for skin treatments in wider geographical areas, and products that are constituents of Islamic medicine (e.g. *Salvadora persica*) historically obtained by Sahrawi in markets peripheral to their nomadic areas. Although these broad procurement patterns reflect those of pre-war nomadic Sahrawi, changes have occurred with displacement and exile to the refugee camps: refugees procure remedies from the former nomadic territories – known as *badyia* – by means of a variety of social networks that have developed since exile and, as well, important products that are in high demand have been commoditized and are today collected by refugees in the *badyia* and sold in the camps or to other (Algerian) traders. This is the case, for example, with the resin of *Acacia tortilis*, the seeds of *Ammodaucus leucotrichus*, the leaves of *Cleome africana*, and the flowering tops of *Teucrium chardonianum*.

**Figure 8:**
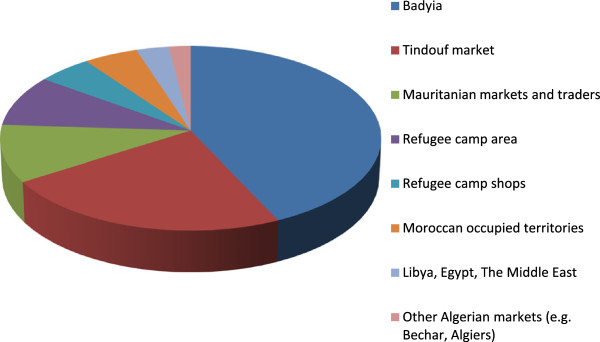
Places where Sahrawi refugees procure plants and vegetal products for cosmetic and skin healing purposes.

The places where Sahrawi refugees procure plants and vegetal products for cosmetic and skin healing purposes are presented in the last column of Table 1 and in Figure 8. More than 40% of these products come from the *badyia*, which includes the strip of Western Sahara under Polisario control (especially its northern areas of Tifariti, Bir Lehlou, and Mehris) and part of northern Mauritania. In nine percent of the cases, products are directly collected from the refugee camps (the environment in and immediately around the refugee camps); this low figure is due to the fact that the Hammada of Tindouf, where refugee camps are located, is relatively poor in biodiversity and thus offers opportunities to collect only a very limited number of species (e.g. *Calotropis procera*, *Pergularia tomentosa*, *Zygophyllum gaetulum*). Sahrawi refugees purchase products in Tindouf market (23% of the cases), in Mauritanian markets and from Mauritanian sellers (10%), in refugee camp shops (five percent), in markets in the occupied territories of Western Sahara (five percent) and, less often, in other remoter markets and places where refugees sometimes travel (e.g. the markets of Béchar or Algiers in two percent of the cases). 

Displaced and migrating populations continuously adapt procurement patterns in order to gain and maintain access to culturally important products. Today some products are sold in the camp shops that opened over the past decade as a result of the ceasefire and the demobilization of Polisario troops, as well as of refugees’ increased access to cash through Spanish civil pensions, remittances, or other productive activities. This, for example, is the case with *henna*, which Sahrawi nomads historically used to obtain from Morocco to the North (Morocco is still amongst the top five growers and exporters in the world) or from Mauritania and Senegal to the South. Nowadays, products of Mauritanian or Algerian origin can be found in many shops in the refugee camps. Other products are still obtained from the same markets at the periphery of the Sahrawi’s customary nomadic areas as in pre-war times: for example, it is reported that, around the middle of the 20th century, Mauritania was the main source of aromatic products for the Reguibat tribe [16], and Caratini [27] (pg 66), in her monograph on the Reguibat, says that they obtain a product known as *tara* (tubercules of *Cyperus rotundus*), described as a ‘kind of nut used by women as a beauty product’, from the agricultural markets of Sudan and the Sahelian regions.

Other products are obtained from as far away as the Middle East; for example, the Sahrawi return from the pilgrimage to Mecca with sandalwood. Sandalwood is mostly produced from wild trees in India and Indonesia, and India accounts for about 90% of sandal oil production in the world [64]. Trade dates back to antiquity, with sandalwood being transported (along with spices, gems, rice, and peacocks) in the first millennium BC from India to the Middle East along the coasts of the Persian Gulf and across the Arabian Desert on dromedaries. When costly products such as sandalwood are involved, Sahrawi refugees are aware of the presence of possible fakes or adulteration in the markets: they say that real sandalwood (*oud legmari*) has a dark color, and fake sandalwood (*oud legmari el haibe*, lit. false sandalwood) is wood of a lighter color to which a colorant is added (indeed, many species of *Santalum*, and also of other genera, are today commercialized as sandalwood).

An interesting case of transnational trade is represented by mastic gum from *Pistacia* species (mainly from *Pistacia lentiscus* L., but also from *Pistacia atlantica* Desf., *Pistacia eurycarpa* Yalt., and *Pistacia palaestina* Bois.). This distinctively flavored resin, commonly known as *mastic* or *mastika*, has been used for more than two thousands years in the Mediterranean and Middle East regions as a chewing gum, a medicinal product, and as a flavoring agent for different foods and drinks [49,65]. It is today used industrially to obtain a variety of products from incense to chewing gum and glue (e.g. Italian *mastice*). Sahrawi refugees buy packets of *tidikt* – as the Sahrawi call mastic – in the markets of Tindouf or Mauritania (e.g. in Zouérat), where they presumably come from France (as indicated from the label ‘made in France’). However, most refugees find them too expensive, and thus only well-off families use them, and less expensive sources are being sought. *Tidikt* is sometimes sent from the refugee camps to emigrants in Spain along with other traditional products such as *Acacia* resin and camel hump. Some Sahrawi traders, mainly living in the Basque Country, have recently started to procure *tidikt* directly from France, where it is allegedly found to be less expensive, and then sell it on to Sahrawi emigrants in Spain, providing evidence of Spanish Sahrawi diaspora networks.

Concerning animal and mineral products, similar procurement patterns are evident: most minerals and animal species are characteristic of the Sahrawi’s nomadic territory and are procured there (animal species are either wild – e.g. fennec, spiny-tailed lizard, darkling beetle – or domesticated – e.g. camel, goat). A few other products are procured in markets (e.g. chameleon), or substituted with more readily available products (e.g. chameleon is substituted with the spiny-tailed lizard, which is perhaps the most common wild animal in the *badyia*). Uses of products obtained from animals no longer present in the *badyia* and not subject to trade have been abandoned. This is, for example, the case with ostrich-derived products, as ostriches have largely disappeared from the area due to drought, hunting, and the effects of Polisario-Morocco war.

## Conclusions

The purpose of the study was to examine Sahrawi refugees’ traditional ethnobiological knowledge and practices and the changes that occurred to these and to product procurement with forced displacement. This study identified 55 plant species and 18 animal or mineral products used by Sahrawi refugees for cosmetic, aromatizing, and skin healing purposes, and gave an account of the use and importance of these products in Sahrawi culture and practices and of the means by which refugees procure them. Results show that refugees still widely use traditional products for cosmetic and skin healing purposes; that these products play an important role in their everyday practices, wellbeing, and cultural and social identity; and that procurement patterns were adapted with forced displacement. Refugees developed new procurement networks in order to maintain access to plants from the former nomadic territories – the *badyia* – and trade networks have been used, adapted, or created to satisfy refugees’ demand for these products.

These results support the idea that refugees, even when constrained to one of the least productive natural environments in the world so that they are largely dependent on aid for survival, are not simply passive recipients of national and international aid, but rather struggle in many ways to maintain and recover practices based on traditionally used vegetal, animal, and mineral products. By taking products used for cosmetic and skin healing purposes as a case study, the current findings add to a growing literature on refugees’ agency and on the ethnobiological practices of migrating and displaced people, and contribute to the preservation of Sahrawi ethnobiological knowledge. We suggest that more research should be carried out on traditional cosmetic and aromatizing practices among different world populations, as well as on the ethnobiological practices of displaced populations and the changes provoked by displacement.

## Competing interests

The authors declare that they have no competing interests.

## Authors’ contributions

GV designed the methods approach, carried out field work, composed the literature review and drafted the manuscript. PK participated in the study design, carried out part of the fieldwork and worked on the graphics. VZ participated in the study design, and helped to analyze the data and to draft the manuscript. All authors read and approved the final manuscript.
